# Complete and Draft Genome Sequences of 52 *Bacillus* and *Priestia* Strains Isolated from West African Fermentations and 26 Reference Strains from a Public Culture Collection

**DOI:** 10.1128/mra.00394-23

**Published:** 2023-06-20

**Authors:** Emma Schack Wiedenbein, Tessa Suzanne Canoy, Yan Hui, Charles Parkouda, Clarisse S. Compaoré, Elmer Ametefe, Mogens Jakobsen, Lene Jespersen, Dennis Sandris Nielsen

**Affiliations:** a Department of Food Science, University of Copenhagen, Copenhagen, Denmark; b Département Technologie Alimentaire, Centre National de la Recherche Scientifique et Technologique, Institut de Recherche en Sciences Appliquées et Technologies, Ouagadougou, Burkina Faso; c Department of Biochemistry, Cell and Molecular Biology, University of Ghana, Accra, Ghana; DOE Joint Genome Institute

## Abstract

The whole genomes of 78 *Bacillus* and *Priestia* strains isolated from West African fermented foods (*n* = 52) or acquired from a public culture collection (*n* = 26) were sequenced using long-read sequencing and assembled into draft (*n* = 32) and complete (*n* = 46) genomes, allowing comparative genomics and taxonomic assignment of these strains with putative uses in fermented foods.

## ANNOUNCEMENT

Fermentation with selected *Bacillus* and *Priestia* species can improve the nutritional, flavor, and safety properties of some food products (1, 2). Whole-genome sequencing (WGS) was conducted on 26 strains from the Deutsche Sammlung von Mikroorganismen und Zellkulturen (DSMZ) culture collection and 52 strains isolated from West African fermented food products (3, 4) (Table 1). This represents a significant contribution to the number of available *Bacillus* and *Priestia* whole genomes in public databases, which will enhance future research on their applications in food fermentations.

**TABLE 1:** Accession details and characteristics of the 78 sequenced *Bacillus* and *Priestia* genomes^*a*^

Isolate (African ID)	Isolation site (source)	Taxonomy (reference strain)	dDDH (%)	ANIm (%)	GenBank accession no.	SRA accession no.	Assembly size (Mbp)	No. of raw reads	G+C content (%)	Coverage (×)	No. of contigs	*N*_50_ (Mbp)^*b*^	Genome status	BUSCO value^*c*^					
														C (%)	S (%)	D (%)	F (%)	M (%)	*n*
DSM 13019	Denmark (boiled hay)	Bacillus subtilis (ATCC 6051)	86.5	98.53	CP120621	SRR23725181	4.04	1.17 × 10^9^	43.61	288.22	1	4.04	Complete	99.3	99.1	0.2	0.4	0.3	450
**DSM 10^T^**	Unknown (“Marburg”)	B. subtilis (ATCC 6051 = DSM 10)	100	99.99	CP120680, CP120681	SRR23725215	4.30	4.57 × 10^8^	43.35	106.29	2	4.22	Complete	99.1	98.9	0.2	0.9	0	450
DSM 1670	Unknown (tropical soil)	Priestia megaterium (ATCC 14581)	94.7	99.44	JARPAC000000000	SRR23725159	5.57	6.36 × 10^8^	37.80	114.04	11	5.24	Draft	99.3	98	1.3	0.7	0	450
DSM 26543	Plön, Germany (mouse lung)	B. licheniformis (ATCC 14580)	92.8	99.21	JAROZY000000000	SRR23725210	4.43	1.91 × 10^8^	45.95	43.22	3	4.38	Draft	65.4	64.7	0.7	26.7	7.9	450
DSM 1969	Unknown (soil)	B. licheniformis (ATCC 14580)	91.7	99.06	JAROZZ000000000	SRR23725213	4.36	4.57 × 10^8^	45.90	104.76	2	4.36	Draft	57.8	57.8	0	31.6	10.6	450
DSM 23521	Unknown	B. subtilis (ATCC 6051)	100	99.99	CP120612, CP120613	SRR23725211	4.21	6.79 × 10^8^	43.47	161.26	2	4.20	Complete	99.1	98.9	0.2	0.9	0	450
DSM 3257	Unknown	B. subtilis (ATCC 6051)	89.4	98.84	JAROZV000000000	SRR23725202	4.34	7.24 × 10^8^	43.45	166.79	6	3.70	Draft	99.1	98.7	0.4	0.9	0	450
DSM 103869	Turin, Italy (cleanroom facility)	B. pumilus^*d*^ (ATCC 7061)	36.5^*e*^	89.04^*e*^	CP120623, CP120624, CP120625	SRR23725214	3.85	1.75 × 10^8^	41.26	45.35	3	3.79	Complete	94.9	94.7	0.2	3.8	1.3	450
DSM 3641	Unknown (soil)	*P. megaterium* (ATCC 14581)	76.2	99.95	JAROZU000000000	SRR23725201	5.60	3.05 × 10^8^	37.77	54.42	7	5.02	Draft	96.9	95.6	1.3	2.9	0.2	450
DSM 1090	Unknown (soil)	B. subtilis (ATCC 6051)	100	99.98	CP120622	SRR23725203	4.14	9.03 × 10^8^	43.79	218.03	1	4.14	Complete	98.9	98.7	0.2	1.1	0	450
DSM 1794	Unknown (soil)	*B. pumilus* (ATCC 7061)	67.9^*e*^	96.26	JARPAA000000000	SRR23725152	3.80	4.04 × 10^8^	41.57	106.38	3	3.79	Draft	100	99.8	0.2	0	0	450
DSM 766	Atlantic Ocean (manganese nodule)	*B. pumilus*^*f*^ (ATCC 7061)	45^*e*^	91.68^*e*^	JARZFY000000000	SRR23725197	3.80	5.87 × 10^8^	41.70	154.54	2	3.72	Draft	99.1	98.9	0.2	0.9	0	450
**DSM 13^T^**	Unknown	B. licheniformis (ATCC 14580 = DSM 13)	100	99.98	JARPAE000000000	SRR23725192	4.24	4.91 × 10^8^	46.21	115.81	2	4.23	Draft	98.6	98.2	0.4	0.9	0.5	450
DSM 603	Unknown (septic wound)	B. licheniformis^*g*^ (ATCC 14580)	58.1^*e*^	94.71^*e*^	JARZFZ000000000	SRR23725198	4.48	5.48 × 10^8^	45.94	122.31	2	4.45	Draft	98.8	98.4	0.4	0.9	0.3	450
DSM 1804	Unknown	*P. megaterium*^*h*^ (ATCC 14581)	65.4^*e*^	96.96	CP120615, CP120616, CP120617, CP120618, CP120619, CP120620	SRR23725153	5.31	1.42 × 10^9^	38.10	268.21	6	5.11	Complete	98.9	97.3	1.6	1.1	0	450
DSM 28591	Freising, Germany (caecal content mouse)	B. licheniformis^*i*^ (ATCC 14580)	58^*e*^	94.64^*e*^	CP120610	SRR23725208	4.39	6.54 × 10^8^	45.90	148.87	1	4.39	Complete	98.4	98	0.4	1.1	0.5	450
DSM 21393	Tatarstan, Russian Federation (potato tubers)	B. subtilis (ATCC 6051)	89.1	98.8	CP120614	SRR23725212	4.10	4.33 × 10^8^	43.85	105.54	1	4.10	Complete	83.6	83.6	0	12	4.4	450
DSM 3228	Unknown	*P. megaterium* (ATCC 14581)	91.2	99.09	JAROZW000000000	SRR23725204	5.72	4.95 × 10^8^	37.71	86.52	13	5.16	Draft	97.3	96	1.3	2.2	0.5	450
DSM 28096	Egypt (soil)	B. licheniformis^*j*^ (ATCC 14580)	19.2^*e*^	85.55^*e*^	CP120611	SRR23725209	4.10	3.90 × 10^8^	43.85	95.04	1	4.10	Complete	84.9	84.9	0	10.9	4.2	450
DSM 5611	Unknown (corn starch)	B. subtilis (ATCC 6051)	86.7	98.55	CP120602, CP120603	SRR23725199	4.29	1.62 × 10^8^	43.46	37.84	2	4.23	Complete	95.5	95.1	0.4	3.8	0.7	450
DSM 2894	Unknown (soil)	*P. megaterium* (ATCC 14581)	68.6^*e*^	96.40	JAROZX000000000	SRR23725207	6.17	9.69 × 10^8^	37.47	157.00	15	4.97	Draft	98.9	97.8	1.1	1.1	0	450
DSM 492	USA	*B. pumilus*^*k*^ (ATCC 7061)	60.2^*e*^	95.04^*e*^	JAROZT000000000	SRR23725200	3.75	5.19 × 10^8^	42.11	138.40	3	3.68	Draft	57.1	56.9	0.2	32.4	10.5	450
DSM 17641	Mikkeli, Finland (paperboard)	*P. megaterium* (ATCC 14581)	72.3	96.83	JARPAB000000000	SRR23725156	5.85	1.11 × 10^9^	37.76	189.27	10	5.21	Draft	98.9	97.1	1.8	0.9	0.2	450
DSM 13835	Finland (indoor air)	*B. pumilus* (ATCC 7061)	75.8	97.31	JARPAD000000000	SRR23725170	3.81	2.57 × 10^8^	41.72	67.45	3	3.79	Draft	68.4	68.4	0	23.1	8.5	450
**DSM 32^T^**	Unknown	*P. megaterium* (ATCC 14581 = DSM 32)	100	99.99	CP120604, CP120605, CP120606, CP120607, CP120608	SRR23725205	5.70	7.22 × 10^8^	37.83	126.77	5	5.29	Complete	98.2	96.9	1.3	1.6	0.2	450
DSM 319	Unknown	*P. megaterium* (ATCC 14581)	86.1	98.42	CP120609	SRR23725206	5.10	2.81 × 10^8^	38.15	55.14	1	5.10	Complete	98	96.7	1.3	1.8	0.2	450
PRO31 (A5)	Nyankpala, Ghana^*l*^ (kantong)	B. safensis^*m*^ (FO-36b)	18.2^*e*^	85.1^*e*^	CP120584, CP120585	SRR23725176	4.27	8.28 × 10^8^	43.19	193.70	2	4.20	Complete	99.3	99.1	0.2	0.7	0	450
PRO33 (B11)	Nyankpala, Ghana^*n*^ (kantong)	*B. safensis* (FO-36b)	91.0	96.4	CP121752	SRR23725175	3.77	4.65 × 10^8^	41.45	123.36	1	3.77	Complete	63.6	63.6	0	25.3	11.1	450
PRO35 (C3)	Nyankpala, Ghana^*l*^ (kantong)	B. amyloliquefaciens^*o*^ (DSM 7)	21.2^*e*^	84.5^*e*^	CP120578, CP120579, CP120580, CP120581, CP120582, CP120583	SRR23725174	4.03	7.44 × 10^8^	43.35	184.61	6	3.95	Complete	99.3	99.1	0.2	0.7	0	450
PRO36 (C4)	Nyankpala, Ghana^*l*^ (kantong)	B. velezensis (NRRL B-41580)	87.7	98.7	JAROZK000000000	SRR23725173	4.09	6.68 × 10^8^	46.48	163.58	2	4.08	Draft	68.9	68.9	0	24.4	6.7	450
PRO37 (C2)	Nyankpala, Ghana^*l*^ (kantong)	*B. safensis* (FO-36b)	77.4	97.5	JAROZJ000000000	SRR23725172	3.78	3.30 × 10^8^	41.67	87.08	3	3.73	Draft	98.2	98	0.2	1.8	0	450
PRO38 (C6)	Nyankpala, Ghana^*n*^ (kantong)	*B. velezensis* (NRRL B-41580)	87.7	98.7	CP121750, CP121751	SRR23725171	4.08	6.60 × 10^8^	46.50	161.56	2	4.08	Complete	72.2	72.2	0	22	5.8	450
PRO40 (D7)	Nyankpala, Ghana^*l*^ (kantong)	*B. amyloliquefaciens* (DSM 7)	85.0	98.3	JAROZI000000000	SRR23725169	3.84	6.38 × 10^8^	46.03	166.18	2	3.83	Draft	99.3	99.3	0	0.2	0.5	450
PRO41 (S53)	Nyankpala, Ghana^*l*^ (kantong)	*B. velezensis* (NRRL B-41580)	93.0	99.2	JAROZH000000000	SRR23725168	4.17	8.94 × 10^8^	46.05	214.54	2	4.16	Draft	99.1	99.1	0	0.9	0	450
PRO45 (S10b1)	Nyankpala, Ghana^*l*^ (kantong)	B. subtilis (ATCC 6051)	83.7	98.2	CP121747, CP121748, CP121749	SRR23725167	3.98	7.14 × 10^8^	43.43	179.63	3	3.96	Complete	66.4	66.2	0.2	25.6	8	450
PRO49 (E4b)	Nyankpala, Ghana^*n*^ (kantong)	B. subtilis (ATCC 6051)	81.0	97.9	CP121743, CP121744, CP121745, CP121746	SRR23725166	4.15	7.63 × 10^8^	43.35	183.73	4	4.11	Complete	72	71.8	0.2	20	8	450
PRO51 (E15)	Nyankpala, Ghana^*n*^ (kantong)	B. subtilis (ATCC 6051)	81.7	98.0	CP121740, CP121741, CP121742	SRR23725165	3.97	3.21 × 10^8^	43.26	80.75	3	3.94	Complete	46.7	46.7	0	34	19.3	450
PRO52 (T3)	Nyankpala, Ghana^*n*^ (kantong)	B. subtilis (ATCC 6051)	83.1	98.1	CP121736, CP121737, CP121738, CP121739	SRR23725164	3.97	8.97 × 10^8^	43.61	226.01	4	3.93	Complete	72.2	71.8	0.4	21.3	6.5	450
PRO53 (T6a)	Nyankpala, Ghana^*n*^ (kantong)	B. subtilis (ATCC 6051)	87.6	98.6	CP120577	SRR23725163	4.11	9.43 × 10^8^	43.72	229.33	1	4.11	Complete	99.8	99.6	0.2	0.2	0	450
PRO55 (U3b)	Nyankpala, Ghana^*l*^ (kantong)	B. subtilis (ATCC 6051)	87.7	98.6	CP121735	SRR23725162	4.09	1.04 × 10^9^	43.55	254.65	1	4.09	Complete	48.7	48.7	0	32.7	18.6	450
PRO56 (U2a2)	Nyankpala, Ghana^*l*^	B. subtilis (ATCC 6051)	85.2	98.4	CP120574, CP120575, CP120576	SRR23725161	4.07	8.81 × 10^8^	43.50	216.56	3	4.00	Complete	97.3	97.1	0.2	2	0.7	450
PRO57 (U13a)	Nyankpala, Ghana^*l*^	B. subtilis (ATCC 6051)	81.1	97.9	JAROZG000000000	SRR23725160	4.00	5.26 × 10^7^	43.63	13.13	8	1.05	Draft	55.8	55.6	0.2	27.1	17.1	450
PRO58 (V7a)	Nyankpala, Ghana^*n*^ (kantong)	B. subtilis (ATCC 6051)	83.9	98.2	CP121732, CP121733, CP121734	SRR23725158	3.98	7.32 × 10^8^	43.45	184.19	3	3.95	Complete	67.3	67.1	0.2	24.9	7.8	450
PRO59 (V11b)	Nyankpala, Ghana^*n*^ (kantong)	B. subtilis (ATCC 6051)	83.9	98.2	CP121731	SRR23725138	3.95	1.31 × 10^9^	43.46	331.89	1	3.95	Complete	70.2	70	0.2	22	7.8	450
PRO61 (W10a)	Nyankpala, Ghana^*l*^ (kantong)	B. subtilis (ATCC 6051)	85.3	98.4	CP121727, CP121728, CP121729, CP121730	SRR23725139	4.02	1.49 × 10^9^	43.60	371.08	4	4.00	Complete	80.2	80.2	0	14.9	4.9	450
PRO64 (W4a)	Nyankpala, Ghana^*n*^ (kantong)	B. subtilis^*p*^ (ATCC 6051)	80.4	97.9	CP121724, CP121725, CP121726	SRR23725140	4.11	6.13 × 10^8^	43.55	148.97	3	4.04	Complete	71.1	70.7	0.4	22.2	6.7	450
PRO67 (SOW2a)	Nyankpala, Ghana^*n*^ (kantong)	*B. velezensis* (NRRL B-41580)	92.1	99.1	JAROZF000000000	SRR23725154	4.31	5.19 × 10^8^	45.68	120.39	8	3.72	Draft	84.4	84.4	0	12.9	2.7	450
PRO72 (Y4b)	Nyankpala, Ghana^*l*^ (kantong)	B. subtilis (ATCC 6051)	81.2	97.9	CP121720, CP121721, CP121722, CP121723	SRR23725155	4.02	5.53 × 10^8^	43.17	137.51	4	3.97	Complete	49.8	49.8	0	28.9	21.3	450
PRO73 (Y3a1)	Nyankpala, Ghana^*l*^ (kantong)	B. subtilis (ATCC 6051)	80.7	97.9	CP121717, CP121718, CP121719	SRR23725141	4.07	8.06 × 10^8^	43.56	198.04	3	4.01	Complete	67.3	67.1	0.2	25.6	7.1	450
PRO74 (Y5)	Nyankpala, Ghana^*l*^ (kantong)	*B. amyloliquefaciens*^*q*^ (DSM 7)	20.7^*e*^	84.39^*e*^	CP121713, CP121714, CP121715, CP121716	SRR23725142	3.99	7.67 × 10^8^	43.23	192.00	4	3.97	Complete	35.3	35.3	0	37.3	27.4	450
PRO76 (G15)	Nyankpala, Ghana^*n*^ (kantong)	*B. velezensis* (NRRL B-41580)	90.8	99.0	CP120571, CP120572, CP120573	SRR23725143	4.17	8.60 × 10^8^	45.88	206.32	3	4.16	Complete	83.1	82.9	0.2	12.9	4	450
PRO77 (G12)	Nyankpala, Ghana^*n*^ (kantong)	*B. velezensis* (NRRL B-41580)	88.0	98.7	JAROZE000000000	SRR23725144	4.10	6.72 × 10^8^	46.45	163.97	2	4.09	Draft	76	76	0	18.2	5.8	450
PRO81 (G17)	Nyankpala, Ghana^*n*^ (kantong)	*B. velezensis* (NRRL B-41580)	79.2	97.7	CP121712	SRR23725157	3.93	6.94 × 10^8^	46.48	176.93	1	3.93	Complete	73.3	73.3	0	20	6.7	450
PRO83 (G5)	Nyankpala, Ghana^*n*^ (kantong)	*B. amyloliquefaciens*^*r*^ (DSM 7)	55.8^*e*^	94.29^*e*^	JAROZD000000000	SRR23725145	4.15	6.68 × 10^8^	45.98	161.13	2	4.11	Draft	98.9	98.9	0	1.1	0	450
PRO84 (G2)	Nyankpala, Ghana^*n*^ (kantong)	B. subtilis (ATCC 6051)	81.2	97.9	CP121708, CP121709, CP121710, CP121711	SRR23725146	4.04	5.21 × 10^8^	43.19	129.15	4	3.99	Complete	49.8	49.8	0	33.1	17.1	450
PRO91 (03)	Navrongo, Ghana^*s*^ (kantong)	*B. velezensis* (NRRL B-41580)	87.3	98.7	JAROZC000000000	SRR23725147	4.12	6.94 × 10^8^	46.42	168.54	2	4.11	Draft	71.1	71.1	0	21.3	7.6	450
PRO93 (042)	Navrongo, Ghana^*s*^ (kantong)	B. subtilis (ATCC 6051)	80.4	97.9	CP121705, CP121706, CP121707	SRR23725148	4.07	4.82 × 10^8^	43.58	118.34	3	4.01	Complete	70.4	70.2	0.2	22.4	7.2	450
PRO96 (243)	Navrongo, Ghana^*s*^ (kantong)	*B. velezensis* (NRRL B-41580)	79.0	97.7	CP121704	SRR23725149	3.89	1.43 × 10^8^	46.55	36.65	1	3.89	Complete	69.3	69.3	0	23.1	7.6	450
PRO97 (481)	Navrongo, Ghana^*s*^ (kantong)	*B. velezensis* (NRRL B-41580)	90.8	99.0	JAROZB000000000	SRR23725150	4.16	9.60 × 10^8^	45.88	230.51	5	4.16	Draft	85.1	85.1	0	10.9	4	450
PRO99 (DP4)	Navrongo, Ghana^*s*^ (kantong)	B. subtilis (ATCC 6051)	83.5	98.2	CP121701, CP121702, CP121703	SRR23725151	3.96	3.69 × 10^8^	43.43	92.96	3	3.95	Complete	67.8	67.6	0.2	21.3	10.9	450
PRO102 (DP2)	Navrongo, Ghana^*s*^ (kantong)	*B. velezensis* (NRRL B-41580)	92.1	99.2	JAROZS000000000	SRR23725196	4.31	4.87 × 10^8^	45.69	113.16	7	3.72	Draft	86.2	86.2	0	11.8	2	450
PRO103 (DP15)	Navrongo, Ghana^*s*^ (kantong)	*B. velezensis* (NRRL B-41580)	93.1	99.2	JAROZR000000000	SRR23725195	4.01	5.68 × 10^8^	46.53	141.74	2	4.00	Draft	99.1	99.1	0	0.9	0	450
PRO104 (DP14)	Navrongo, Ghana^*s*^ (kantong)	P. aryabhattai (JCM 13839)	80.4	97.8	JAROZQ000000000	SRR23725194	5.67	2.06 × 10^8^	37.94	36.24	10	5.16	Draft	97.8	96.9	0.9	2.2	0	450
PRO107 (FP3)	Navrongo, Ghana^*s*^ (kantong)	*B. altitudinis* (DSM 21631)	82.4	98.1	JAROZP000000000	SRR23725193	3.85	3.49 × 10^8^	41.34	90.59	3	3.79	Draft	89.1	88.7	0.4	8.2	2.7	450
PRO108 (FP9)	Navrongo, Ghana^*s*^ (kantong)	*B. velezensis* (NRRL B-41580)	92.0	99.1	JAROZO000000000	SRR23725191	4.31	4.02 × 10^8^	45.69	93.20	9	3.72	Draft	84.7	84.7	0	12	3.3	450
PRO109 (FP1)	Navrongo, Ghana^*s*^ (kantong)	B. paralicheniformis (KJ-16)	90.1	98.9	CP120601	SRR23725190	4.62	8.20 × 10^8^	45.55	177.63	1	4.62	Complete	98.2	97.8	0.4	1.3	0.5	450
PRO112 (B83)	Mansila, Burkina Faso^*t*^ (maari)	B. subtilis (ATCC 6051)	87.6	98.6	CP120600	SRR23725189	4.28	6.47 × 10^8^	43.71	151.13	1	4.28	Complete	99.5	99.3	0.2	0.4	0.1	450
PRO113 (B303)	Gorgadji, Burkina Faso^*u*^ (maari)	*P. aryabhattai* (JCM 13839)	79.9	97.8	JAROZN000000000	SRR23725188	5.71	3.48 × 10^8^	37.88	60.93	11	5.13	Draft	98	96.4	1.6	2	0	450
PRO114 (B313)	Gorgadji, Burkina Faso^*u*^ (maari)	*B. safensis* (FO-36b)	77.6	97.5	CP120599	SRR23725187	3.77	9.90 × 10^8^	41.65	262.42	1	3.77	Complete	96	95.8	0.2	3.3	0.7	450
PRO115 (B56)	Mansila, Burkina Faso^*t*^ (maari)	B. subtilis (ATCC 6051)	99.9	100.0	CP120598	SRR23725186	4.14	1.85 × 10^8^	43.79	44.63	1	4.14	Complete	97.5	97.3	0.2	2	0.5	450
PRO116 (B360)	Mansila, Burkina Faso^*t*^ (maari)	P. flexa (NBRC 15715)	94.5	99.4	CP120590, CP120591, CP120592, CP120593, CP120594, CP120595, CP120596, CP120597	SRR23725185	3.99	7.48 × 10^8^	37.94	187.20	8	3.91	Complete	98.3	97.6	0.7	1.3	0.4	450
PRO117 (B3)	Toulfé, Burkina Faso^*v*^ (maari)	B. subtilis (ATCC 6051)	83.9	98.3	JAROZM000000000	SRR23725184	4.53	5.53 × 10^8^	42.90	122.13	4	4.40	Draft	98.2	94.4	3.8	1.8	0	450
PRO118 (B222)	Toulfé, Burkina Faso^*v*^ (maari)	B. subtilis (ATCC 6051)	79.1	97.7	CP121759, CP121760, CP121761, CP121762	SRR23725183	4.44	4.87 × 10^8^	42.68	109.78	4	4.39	Complete	48.9	48.9	0	33.6	17.5	450
PRO119 (B122)	Mansila, Burkina Faso^*t*^ (maari)	*P. aryabhattai* (JCM 13839)	80.1	97.8	JAROZL000000000	SRR23725182	5.68	4.72 × 10^8^	37.92	83.19	11	5.13	Draft	99.2	97.6	1.6	0.9	0.1	450
PRO120 (B163)	Toulfé, Burkina Faso^*v*^ (maari)	B. subtilis (ATCC 6051)	85.1	98.3	CP120588, CP120589	SRR23725180	4.19	7.42 × 10^8^	43.33	177.06	2	4.17	Complete	98.9	98.7	0.2	0.9	0.2	450
PRO121 (B215)	Toulfé, Burkina Faso^*v*^ (maari)	B. subtilis (ATCC 6051)	84.6	98.3	CP120586, CP120587	SRR23725179	4.40	8.73 × 10^8^	43.20	198.47	2	4.39	Complete	96.9	96.7	0.2	2.4	0.7	450
PRO122 (B18)	Toulfé, Burkina Faso^*v*^ (maari)	B. subtilis (ATCC 6051)	79.7	97.8	CP121756, CP121757, CP121758	SRR23725178	4.49	1.05 × 10^9^	42.66	234.54	3	4.47	Complete	48.7	48.7	0	32.2	19.1	450
PRO134 (U6b)	Nyankpala, Ghana^*l*^ (kantong)	B. subtilis (ATCC 6051)	83.7	98.18	CP121753, CP121754, CP121755	SRR23725177	3.97	4.91 × 10^8^	43.45	123.67	3	3.95	Complete	69.7	69.3	0.4	22	8.3	450

a Six DSMZ strains were sequenced previously using sequencing technologies other than ONT, namely, DSM 10 (5), DSM 13 (6, 7), DSM 319 (8), DSM 603 (9), DSM 1804 (8), and DSM 32 (10), while DSM 32 was previously sequenced using ONT (11). Five West African strains (PRO31, PRO35, PRO64, PRO74, and PRO83) were previously taxonomically assigned using 16S rRNA or *gyrA* gene sequencing (3). For strains with previous taxonomic assignment (all DSMZ isolates and PRO31, PRO35, PRO64, PRO74, and PRO83), the dDDH and ANIm value matches with the reference genome of the previously assigned species are indicated. The superscript T denotes type strains (in bold).

b The *N*_50_ value denotes the length (Mbp) of the contig that represents 50% of the total assembled genome length (Mbp).

c BUSCO notation: C complete, S complete and single copy, D complete and duplicated, F fragmented, M missing, *n* total number of BUSCO groups searched.

d Acquired as *B. pumilus* but more closely related to *B. altitudinis* (reference genome DSM 21631; dDDH, 84.8%; ANIm, 98.35%).

e Value is below the species threshold (<70% dDDH or <96% ANIm).

f Acquired as *B. pumilus* but more closely related to *B. safensis* (reference genome FO-36b; dDDH, 77.7%; ANIm, 97.5%).

g Acquired as B. licheniformis but more closely related to *B. paralicheniformis* (reference genome KJ-16; dDDH, 94.2%; ANIm, 97.5%).

h Acquired as *P. megaterium* but also closely related to *P. aryabhattai* (reference genome JCM 13839; dDDH, 73.7%; ANIm, 95.81%).

i Acquired as B. licheniformis but more closely related to *B. paralicheniformis* (reference genome KJ-16; dDHH, 92.2%; ANIm, 99.1%).

j Acquired as B. licheniformis but more closely related to B. subtilis (reference genome ATCC 6051; dDDH, 89.1%; ANIm, 98.8%).

k Acquired as *B. pumilus*; most closely related to *B. pumilus*, but the dDDH and ANIm scores (see above) are below the species threshold limits.

l Latitude 9.40224, longitude −0.98356.

m Classified as *B. safensis* by *gyrA* sequencing (3) but more closely related to B. subtilis (reference genome ATCC 6051; dDDH, 85.3%; ANIm, 98.35%).

n Latitude 9.40224, longitude −0.98181.

o Classified as *B. amyloliquefaciens* by *gyrA* sequencing (3) but more closely related to B. subtilis (reference genome ATCC 6051; dDDH, 86%; ANIm, 98.4%).

p Previously classified as B. subtilis by 16S rRNA sequencing (3).

q Classified as *B. amyloliquefaciens* by 16S rRNA sequencing (3) but more closely related to B. subtilis (reference genome ATCC 6051; dDDH, 76.6%; ANIm, 97.4%).

r Classified as *B. amyloliquefaciens* by 16S rRNA sequencing (3) but more closely related to *B. velezensis* (reference genome NRRL B-41580; dDDH, 91.1%; ANIm, 99.1%).

s Latitude 10.86604, longitude −1.07921.

t Latitude 13.17000, longitude 0.63000.

u Latitude 14.03289, longitude −0.51976.

v Latitude 13.64717, longitude −1.62280.

Purified cryostocks (–80°C; brain heart infusion [BHI] broth [HiMedia, Germany] with 20% glycerol) were repropagated in BHI broth and incubated overnight at 30°C at 200 rpm. Cells were collected from overnight cultures by two-step centrifugation (3,900 × *g* for 10 min, decanting, addition of 1 mL 0.9% NaCl solution, and 13,800 × *g* for 3 min). Genomic DNA (gDNA) was extracted from bacterial pellets using the bead-beat micro AX Gravity kit following the instructions of the manufacturer (A&A Biotechnology, Poland; catalog number 106-100). The total gDNA was quantified using a Qubit fluorometer (Invitrogen; 1× double-stranded DNA [dsDNA] high-sensitivity and broad-range assay kits; Thermo Fisher, USA). Sequencing libraries were prepared without size selection or shearing, using the Oxford Nanopore Technologies rapid barcoding kit (SQK-RBK004) and rapid barcoding protocol version RBK_9054_v2_revAE_14Aug2019, following the instructions of the manufacturer. The libraries were loaded onto a MinION flow cell and sequenced on a GridION benchtop device for 72 h using standard settings.

Sequencing data were base called in high-accuracy mode, adapter trimmed using MinKNOW and Guppy (releases 6.1.5, 6.07, 6.06, and 5.1.13), and *de novo* assembled using an in-house workflow (https://github.com/yanhui09/ONTrapid). Briefly, high-quality reads (Q > 10; minimum length, >2,000 bp) were extracted using NanoFilt version 2.8.0 for assembly using Flye version 2.9.1 (12). The generated contigs were polished using two rounds of Racon version 1.5.0 (13) and one round of Medaka version 1.6.0 with the model r941_min_hac_g507. The BUSCO version 5.4.5 tool was used to assess genome completeness (14, 15). Default parameters were used for all software except where otherwise noted.

Genome assembly resulted in 46 complete, closed genomes and 32 draft genomes (Table 1). Digital DNA-DNA hybridization (dDDH) values were determined using the Type Strain Genome Server (TYGS) (https://tygs.dsmz.de) (formula d_4_) (16), with a species delineation threshold of 70% (17). Further confirmation of the identity was provided by calculating the average nucleotide identity based on MUMmer (ANIm) scores, using JSpeciesWS version 3.9.8 (https://jspecies.ribohost.com/jspeciesws/) (18) and a species delineation threshold of 96% (19) (Table 1). For nine DSMZ and four West African isolates, dDDH and ANIm comparisons with the type strain of the previous taxonomic assignment resulted in values below the species threshold (<70% dDDH or <96% ANIm) (Table 1), indicating that their taxonomic assignment may need to be revised.

In summary, long-read sequencing technology allowed us to generate high-quality complete and draft prokaryotic genome sequences and further highlighted the necessity for revising the taxonomy of selected *Bacillus* and *Priestia* strains based on WGS data.

### Data availability.

This WGS project has been deposited in GenBank and SRA under the accession numbers listed in Table 1. The NCBI BioProject accession number is PRJNA941188.

## References

[1] Parkouda C, Nielsen DS, Azokpota P, Ouoba LII, Amoa-Awua WK, Thorsen L, Hounhouigan JD, Jensen JS, Tano-Debrah K, Diawara B, Jakobsen M. 2009. The microbiology of alkaline-fermentation of indigenous seeds used as food condiments in Africa and Asia. Crit Rev Microbiol 35:139–156. doi:10.1080/10408410902793056. 19514912

[2] Biedendieck R, Knuuti T, Moore SJ, Jahn D. 2021. The “beauty in the beast”—the multiple uses of Priestia megaterium in biotechnology. Appl Microbiol Biotechnol 105:5719–5737. doi:10.1007/s00253-021-11424-6. 34263356 PMC8390425

[3] Kpikpi EN, Thorsen L, Glover R, Dzogbefia VP, Jespersen L. 2014. Identification of Bacillus species occurring in kantong, an acid fermented seed condiment produced in Ghana. Int J Food Microbiol 180:1–6. doi:10.1016/j.ijfoodmicro.2014.03.028. 24747716

[4] Parkouda C, Thorsen L, Compaoré CS, Nielsen DS, Tano-Debrah K, Jensen JS, Diawara B, Jakobsen M. 2010. Microorganisms associated with maari, a Baobab seed fermented product. Int J Food Microbiol 142:292–301. doi:10.1016/j.ijfoodmicro.2010.07.004. 20688411

[5] Lilge L, Hertel R, Morabbi Heravi K, Henkel M, Commichau FM, Hausmann R. 2021. Draft genome sequence of the type strain Bacillus subtilis subsp. subtilis DSM10. Microbiol Resour Announc 10:e00158-21. doi:10.1128/MRA.00158-21. 33707332 PMC7953295

[6] Veith B, Herzberg C, Steckel S, Feesche J, Maurer KH, Ehrenreich P, Bäumer S, Henne A, Liesegang H, Merkl R, Ehrenreich A, Gottschalk G. 2004. The complete genome sequence of Bacillus licheniformis DSM13, an organism with great industrial potential. J Mol Microbiol Biotechnol 7:204–211. doi:10.1159/000079829. 15383718

[7] Rey MW, Ramaiya P, Nelson BA, Brody-Karpin SD, Zaretsky EJ, Tang M, de Leon A, Xiang H, Gusti V, Clausen IG, Olsen PB, Rasmussen MD, Andersen JT, Jørgensen PL, Larsen TS, Sorokin A, Bolotin A, Lapidus A, Galleron N, Ehrlich SD, Berka RM. 2004. Complete genome sequence of the industrial bacterium Bacillus licheniformis and comparisons with closely related Bacillus species. Genome Biol 5:R77. doi:10.1186/gb-2004-5-10-r77. 15461803 PMC545597

[8] Eppinger M, Bunk B, Johns MA, Edirisinghe JN, Kutumbaka KK, Koenig SSK, Creasy HH, Rosovitz MJ, Riley DR, Daugherty S, Martin M, Elbourne LDH, Paulsen I, Biedendieck R, Braun C, Grayburn S, Dhingra S, Lukyanchuk V, Ball B, Ul-Qamar R, Seibel J, Bremer E, Jahn D, Ravel J, Vary PS. 2011. Genome sequences of the biotechnologically important Bacillus megaterium strains QM B1551 and DSM319. J Bacteriol 193:4199–4213. doi:10.1128/JB.00449-11. 21705586 PMC3147683

[9] Mendo SA, Henriques IS, Correia AC, Duarte JM. 2000. Genetic characterization of a new thermotolerant Bacillus licheniformis strain. Curr Microbiol 40:137–139. doi:10.1007/s002849910028. 10594231

[10] Arya G, Petronella N, Crosthwait J, Carrillo CD, Shwed PS. 2014. Draft genome sequence of Bacillus megaterium type strain ATCC 14581. Genome Announc 2:e01124-14. doi:10.1128/genomeA.01124-14. PMC4241655 25395629

[11] Shwed PS, Crosthwait J, Weedmark K, Hoover E, Dussault F. 2021. Complete genome sequences of Priestia megaterium type and clinical strains feature complex plasmid arrays. Microbiol Resour Announc 10:e00403-21. doi:10.1128/MRA.00403-21. 34236233 PMC8265218

[12] Kolmogorov M, Yuan J, Lin Y, Pevzner PA. 2019. Assembly of long, error-prone reads using repeat graphs. Nat Biotechnol 37:540–546. doi:10.1038/s41587-019-0072-8. 30936562

[13] Vaser R, Sović I, Nagarajan N, Šikić M. 2017. Fast and accurate de novo genome assembly from long uncorrected reads. Genome Res 27:737–746. doi:10.1101/gr.214270.116. 28100585 PMC5411768

[14] Seppey M, Manni M, Zdobnov EM. 2019. BUSCO: assessing genome assembly and annotation completeness. Methods Mol Biol 1962:227–245. doi:10.1007/978-1-4939-9173-0_14. 31020564

[15] Simão FA, Waterhouse RM, Ioannidis P, Kriventseva EV, Zdobnov EM. 2015. BUSCO: assessing genome assembly and annotation completeness with single-copy orthologs. Bioinformatics 31:3210–3212. doi:10.1093/bioinformatics/btv351. 26059717

[16] Meier-Kolthoff JP, Göker M. 2019. TYGS is an automated high-throughput platform for state-of-the-art genome-based taxonomy. Nat Commun 10:2182. doi:10.1038/s41467-019-10210-3. 31097708 PMC6522516

[17] Chun J, Oren A, Ventosa A, Christensen H, Arahal DR, da Costa MS, Rooney AP, Yi H, Xu X-W, De Meyer S, Trujillo ME. 2018. Proposed minimal standards for the use of genome data for the taxonomy of prokaryotes. Int J Syst Evol Microbiol 68:461–466. doi:10.1099/ijsem.0.002516. 29292687

[18] Richter M, Rosselló-Móra R, Oliver Glöckner F, Peplies J. 2016. JSpeciesWS: a Web server for prokaryotic species circumscription based on pairwise genome comparison. Bioinformatics 32:929–931. doi:10.1093/bioinformatics/btv681. 26576653 PMC5939971

[19] Richter M, Rosselló-Móra R. 2009. Shifting the genomic gold standard for the prokaryotic species definition. Proc Natl Acad Sci USA 106:19126–19131. doi:10.1073/pnas.0906412106. 19855009 PMC2776425

